# Genomic reaction norms inform predictions of plastic and adaptive responses to climate change

**DOI:** 10.1111/1365-2656.13707

**Published:** 2022-05-18

**Authors:** Rebekah A. Oomen, Jeffrey A. Hutchings

**Affiliations:** ^1^ Centre for Ecological and Evolutionary Synthesis (CEES), Department of Biosciences University of Oslo Oslo Norway; ^2^ Centre for Coastal Research (CCR) University of Agder Kristiansand Norway; ^3^ Department of Biology Dalhousie University Halifax Nova Scotia Canada; ^4^ Institute of Marine Research, Flødevigen Marine Research Station His Norway

**Keywords:** common‐garden experiment, environmental change, gene expression, genomic forecasting, local adaptation, phenotypic plasticity, RNA sequencing, transcriptomics

## Abstract

1. Genomic reaction norms represent the range of gene expression phenotypes (usually mRNA transcript levels) expressed by a genotype along an environmental gradient. Reaction norms derived from common‐garden experiments are powerful approaches for disentangling plastic and adaptive responses to environmental change in natural populations. By treating gene expression as a phenotype in itself, genomic reaction norms represent invaluable tools for exploring causal mechanisms underlying organismal responses to climate change across multiple levels of biodiversity.
2. Our goal is to provide the context, framework and motivation for applying genomic reaction norms to study the responses of natural populations to climate change.
3. Here, we describe the utility of integrating genomics with common‐garden‐gradient experiments under a reaction norm analytical framework to answer fundamental questions about phenotypic plasticity, local adaptation, their interaction (i.e. genetic variation in plasticity) and future adaptive potential.
4. An experimental and analytical framework for constructing and analysing genomic reaction norms is presented within the context of polygenic climate change responses of structured populations with gene flow. Intended for a broad eco‐evo readership, we first briefly review adaptation with gene flow and the importance of understanding the genomic basis and spatial scale of adaptation for conservation and management of structured populations under anthropogenic change. Then, within a high‐dimensional reaction norm framework, we illustrate how to distinguish plastic, differentially expressed (difference in reaction norm intercepts) and differentially plastic (difference in reaction norm slopes) genes, highlighting the areas of opportunity for applying these concepts.
5. We conclude by discussing how genomic reaction norms can be incorporated into a holistic framework to understand the eco‐evolutionary dynamics of climate change responses from molecules to ecosystems. We aim to inspire researchers to integrate gene expression measurements into common‐garden experimental designs to investigate the genomics of climate change responses as sequencing costs become increasingly accessible.

## Graphical abstract

A genomic reaction norm framework integrates genome‐wide expression patterns with common‐garden‐gradient experiments to answer fundamental questions about phenotypic plasticity, local adaptation, their interaction, and future adaptive potential. Genomic reaction norms can be incorporated into a holistic framework to understand the eco‐evolutionary dynamics of climate change responses from molecules to ecosystems.

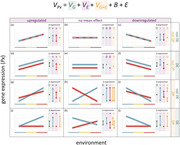

## 1 INTRODUCTION

Global wildlife is experiencing unprecedented threats from anthropogenic sources (IPBES, 2019; United Nations Summit on Biodiversity, 2020). The Intergovernmental Panel on Climate Change (IPCC) highlighted ‘the urgency of prioritizing timely, ambitious and coordinated action to address unprecedented and enduring changes’ (IPCC, 2019). Arguably, the most widespread and urgent danger is that of rising and increasingly variable temperatures (IPCC, 2018, 2019). Mean temperatures have risen 1°C in recent decades and are expected to increase 3–6°C globally, coupled with increased magnitude and frequency of thermal extremes (IPCC, 2018, 2019). The extent of these changes will vary locally, along with a host of other covarying and interacting variables (e.g. precipitation, sea level rise, storm activity; IPCC, 2018). Under these circumstances, populations and species will disperse to more favourable environments (if available), cope with new environmental conditions through phenotypic plasticity, adapt if there is sufficient time and genetic variation to do so or perish via maladaptation to the new environment (Capblancq et al., 2020; Williams et al., 2008). Much attention is focused on predicting these outcomes (e.g. Bay et al., 2017; Brito‐Morales et al., 2018; Lasky et al., 2020; Waldvogel et al., 2020), with the intent of informing effective conservation and management strategies while also highlighting emerging resources for potential sustainable exploitation.

### 1.1 Responses to environmental change often vary geographically and have a genetic basis

As the effects of climate change are expected to vary at local spatial scales (IPCC, 2018), the capacity of populations for plastic and adaptive responses must also be assessed locally (Lasky et al., 2020). Local adaptation produces genetically differentiated populations with traits that can differ in both mean phenotype and phenotypic plasticity (the range of phenotypes expressed by a genotype under different environmental conditions; Bradshaw, 1965). Therefore, spatial environmental heterogeneity can lead to variation in morphology, life history, physiology and behaviour among populations as well as how these phenotypes change in response to changes in the environment (Hutchings, 2011; Oomen & Hutchings, 2015). This genetic variation contributes to the observed global diversity in traits and their plasticities, one consequence of which is that environmental change will affect locally adapted populations differently (Hoffmann et al., 2015). Key parameters affecting responses to climate change across space and time are the genomic basis and spatial scale of variation in adaptive traits. For example, the distribution of adaptive variants relative to environmental variation can facilitate the predictions of population responses of individual traits and ecological dynamics (Bay et al., 2018; Capblancq et al., 2020; Layton et al., 2021; Waldvogel et al., 2020), whereas the genomic architecture of traits under selection affects their evolutionary responses (Bay et al., 2017; Kardos & Luikart, 2021; Oomen et al., 2020).

### 1.2 Genomic reaction norms can link genotype, phenotype and demography

Genomic technologies provide extraordinary opportunities for unravelling the demographic and adaptive processes that form the basis of adaptive evolutionary management (Bernatchez et al., 2017; Hoffmann et al., 2015), notwithstanding challenges in doing so (Coates et al., 2018; Waples & Lindley, 2018). However, most population genetic studies neglect to examine the phenotypic traits potentially undergoing selection (Cushman, 2014; de Villemereuil et al., 2016). More traditional experimental approaches, such as common‐garden and reciprocal transplant experiments, are unmatched for their ability to disentangle genetic and environmental (i.e. plastic) effects on phenotypes, but generally do not seek to identify the specific genomic basis of phenotypic variation (de Villemereuil et al., 2016; Oomen & Hutchings, 2017). As a result, our understanding of the mechanistic links between genotype and phenotype that shape phenotypic variation in time and space is lacking, despite increasingly being cited as important for understanding species responses to environmental change (Lasky et al., 2020).

Distinguishing plastic physiological and behavioural responses to climate from genetic adaptation in wild populations is challenging because phenotypic variation expressed at one developmental or life‐history stage could be attributed either to genetic differences or environmental conditions experienced earlier in life. Distinguishing plastic and evolved responses is critical for understanding the time‐scales and manner in which species are expected to respond to climate change (Crozier & Hutchings, 2014).

**FIGURE 1:**
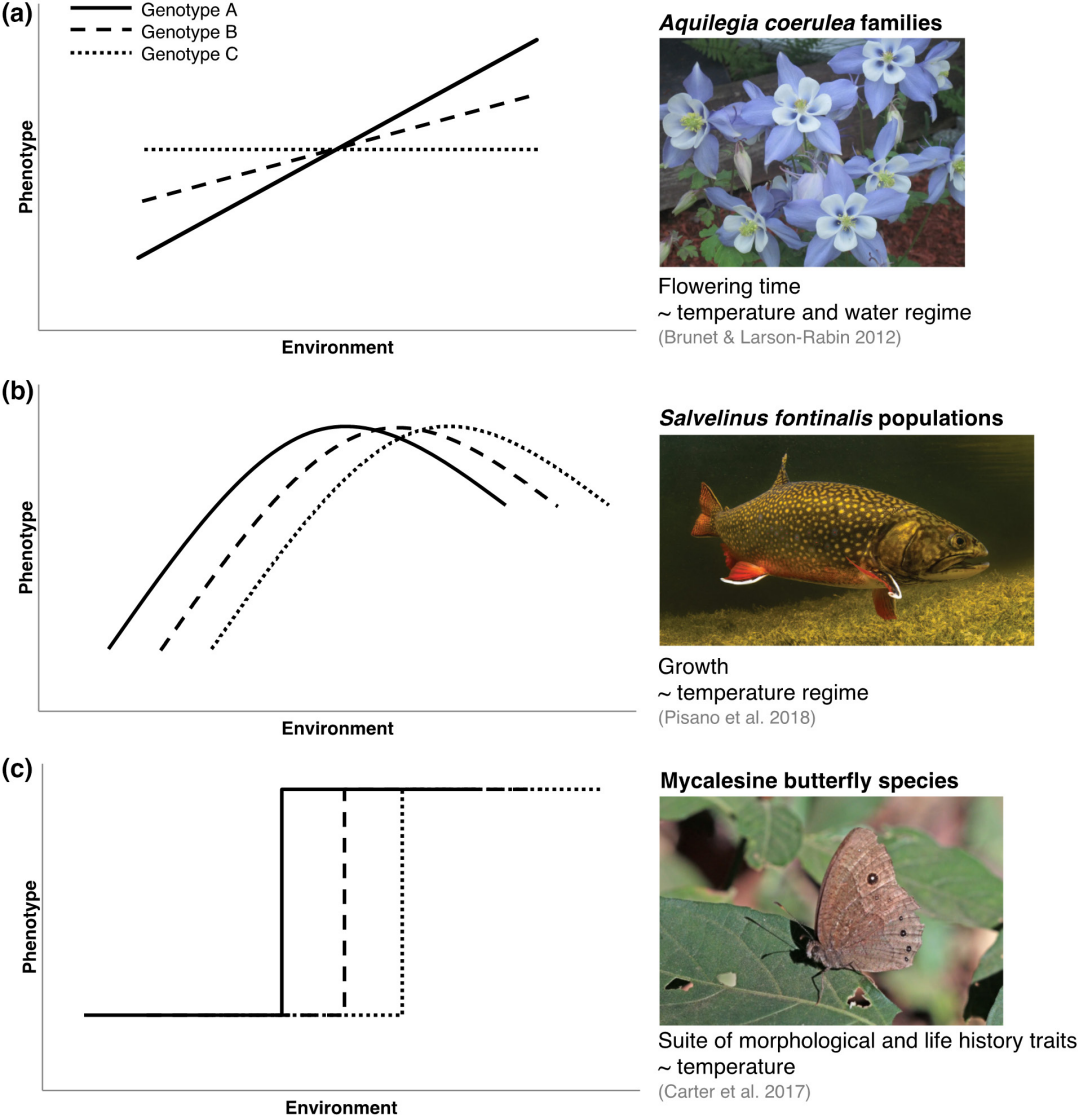
Hypothetical reaction norm variation for three ‘genotypes’ (i.e. families, populations or species) for (a) linear, (b) quadratic and (c) threshold shapes. The examples reflect a variety of organisms, phenotypes and levels of biological organization for which reaction norms are constructed. Photo credits (top to bottom): Rob Duvall; Jay Fleming/US Nat'l Park Svc; Charles J. Sharp

Reaction norms (sensu Woltereck, 1909) are a classic tool for partitioning phenotypic variation into genetic and environmental components as well as their interaction (i.e. *V*_*G*×*E*_; Stearns, 1989; Oomen & Hutchings, 2020). They graphically, mathematically and conceptually represent the range of phenotypes expressed by a genotype, or group of genotypes, along an environmental gradient (Figure 1). Combining such measures with a common‐garden design offers a powerful approach for detecting genetic variation in plasticity (Hutchings, 2011; Oomen & Hutchings, 2015), which is in itself a heritable trait for selection to act upon (Chevin et al., 2010; Lande, 2009; Nussey et al., 2005). Phenotypic variation due to genes, environment and their interaction is reflected by reaction norm intercepts, slopes (or shapes) and differences between slopes (or shapes) respectively (Figure 1; Murren et al., 2014). Therefore, comparing reaction norms among populations reveals whether phenotypic differences are plastic or evolved, thereby affecting the manner and rate at which populations will respond to environmental change.

Reaction norms also inform how plasticity and evolution interact, as alternative hypotheses produce distinct patterns of reaction norm variation among populations. For example, plasticity can promote (via genetic accommodation) or constrain (by buffering the phenotype from selection) adaptive evolution (Crispo & Chapman, 2010; West‐Eberhard, 2003). In the former case, evolution can occur via genetic assimilation (a phenotype ancestrally expressed only during an adaptive plastic response becomes fixed in a new environment) or genetic compensation (maladaptive plasticity is selected against, leading to fixation of the ancestral phenotype; Grether, 2005; Swaegers et al., 2020). Furthermore, comparing reaction norms within populations (e.g. at the family level) produces a measure of standing genetic variation in plasticity, which can be compared among populations to reflect the relative adaptive potentials of their plastic responses to the environment (Harder et al., 2020; Oomen & Hutchings, 2015).

Integrating common‐garden‐gradient experiments with genomics facilitates adaptive and functional insights into genetically based variation in phenotypes and phenotypic responses (Lafuente & Beldade, 2019). Genomics has taken reaction norms into a high‐dimensional era by permitting the examination of molecular phenotypes *en masse*. Genomic reaction norms treat gene expression as a phenotype itself (Aubin‐Horth & Renn, 2009). Gene expression is usually quantified as genome‐wide mRNA transcript abundance (i.e. transcriptomics), though protein expression (i.e. proteomics) can be similarly considered. In this way, genomic reaction norms can be used to link changes in downstream phenotypes and fitness to differences in gene regulation, providing a mechanistic understanding of phenotypic plasticity and how traits and their plasticities evolve (Oomen & Hutchings, 2017). Thus, a hybrid approach that combines reaction norms with genomics is capable of spanning several levels of biological organization and multiple spatial and temporal scales of environmental responses.

Here, we describe a reaction norm framework for integrating genomics with common‐garden‐gradient experiments to answer fundamental questions about phenotypic plasticity, local adaptation, their interaction (i.e. genetic variation in plasticity) and future adaptive potential in the context of climate change. We first briefly review adaptation with gene flow and the importance of understanding the genomic basis and spatial scale of adaptation for conservation and management of structured populations under anthropogenic change. We then propose genomic reaction norms (Aubin‐Horth & Renn, 2009) as a key tool for distinguishing plastic and evolutionary responses to climate and identifying the genomic basis and spatial scale of climate adaptation. Experimental and analytical considerations pertaining to constructing and analysing climate genomic reaction norms are discussed. We conclude by discussing how genomic reaction norms can be incorporated into a holistic framework to understand the eco‐evolutionary dynamics of climate change responses from molecules to ecosystems.

## 2 GENE FLOW AND GENETIC ARCHITECTURE AFFECT THE SPATIAL AND TEMPORAL SCALE OF VARIATION IN CLIMATE RESPONSES

### 2.1 Adaptation can find a way in the face of gene flow

Adaptive divergence is counteracted by the homogenizing the effects of gene flow: swamping of locally adapted alleles and reduced fitness of immigrants (Bulmer, 1972; Lenormand, 2002; Wright, 1931). In addition to the extent of gene flow and the strength of selection, the migration‐selection balance (Felsenstein, 1976) depends on demographic factors, such as population abundance and sex ratio as well as genetic factors, such as frequencies of adaptive variants and genome organization. Despite a large body of empirical and theoretical work describing the interplay between some demographic, genetic and environmental variables (Allendorf et al., 2010; Lande, 1988, 1993; Lowe et al., 2017), there are many unresolved questions that prevent us from predicting the conditions under which local adaptation occurs in nature and the impact of environmental change on wild populations (Bernatchez, 2016; Capblancq et al., 2020; Hansen, Olivieri, et al., 2012).

### 2.2 The genomic basis of adaptation informs eco‐evolutionary responses

A mechanistic understanding of adaptation requires that, at the very least, the specific selective pressure, the phenotypic trait undergoing selection and the genetic basis of that trait be known, in the context of demographic processes. Despite several calls for using integrated approaches to address limitations in our knowledge of these components (e.g. Dalziel et al., 2009; Lowe et al., 2017; Oomen & Hutchings, 2017; Pörtner et al., 2006), progress has been slow for non‐model species. Large‐scale genotyping [e.g. single nucleotide polymorphisms (SNPs)] and next‐generation sequencing (e.g. RAD sequencing, RNA sequencing, long‐ and linked‐read sequencing) technologies coupled with advances in big data handling and bioinformatic software are enabling the characterization of genome‐wide variation in large samples with little or no prior genomic resources (Fuentes‐Pardo & Ruzzante, 2017; Mérot et al., 2020). As a consequence, it is increasingly feasible to isolate the genetic basis of adaptive traits. For example, identifying the genomic basis of parallel adaptation to fresh water in three‐spined sticklebacks *Gasterosteus aculeatus* led to several revelations about the roles of standing genetic variation, non‐coding regulatory elements and genetic and genomic architecture in adaptive evolution (Jones et al., 2012). A recent study in natural populations of nematodes *Caenorhabditis elegans* identified a single point mutation underlying adaptive phenotypic plasticity of a complex trait, matricidal hatching, in response to nutrient levels (Vigne et al., 2021). Genetic and genomic architecture has also emerged as a key mechanism involved in adaptation in the face of gene flow (Nosil et al., 2009; Tigano & Friesen, 2016), trait evolution and recovery under periodic selection (Oomen et al., 2020) and population viability under rapid environmental change (Kardos & Luikart, 2021). Therefore, uncovering the genomic basis of adaptation yields new insights into plasticity and adaptation, with direct relevance for wildlife conservation and management.

### 2.3 The spatial scale of adaptation affects the efficacy of management strategies

Of major concern for wildlife management is the spatial scale at which adaptive variation exists and its distribution relative to spatially heterogeneous selection pressures. Conservation and management practices are frequently implemented geographically (Coates et al., 2018) and often rely on identifying units considered to represent distinct genetic components as targets for conservation (Ryder, 1986; reviewed by Fraser & Bernatchez, 2001). This approach is aimed at preserving intraspecific diversity, which bolsters the adaptive potential of a species and, therefore, its ability to evolve in response to environmental change (Hoffmann & Sgrò, 2011). Intraspecific diversity is also, on average, just as important as species diversity for maintaining broader ecological processes and ecosystem services (Des Roches et al., 2018). Therefore, failing to account for spatially structured adaptive variation in conservation and management plans can erode both the population of interest and ecosystem functioning as a whole. However, governance structures, such as jurisdictional entities responsible for forestry and fisheries management, typically operate on unduly broad scales, making assessment across multiple spatial scales ideal for informing effective and feasible conservation strategies in many cases (Mason & Lashley, 2021; Oppel et al., 2018; Price et al., 2004).

### 2.4 The genetic architecture and spatial scale of adaptation interact

Climate (mal)adaptation is evident on very broad scales, such as between polar, temperate and tropical regions and microgeographic scales, such as across microhabitat (e.g. shade level, elevation, depth) or urbanization gradients, and everything in between. Importantly, even in the absence of reproductive barriers, gene flow depends on geographic barriers, including the distance between the populations relative to their dispersal capabilities (Richardson et al., 2014). Consequently, gene flow usually varies across the range of a species. Because the primary factors driving adaptation are expected to vary with gene flow (Nosil et al., 2009; Tigano & Friesen, 2016), different genomic architectures might underlie adaptation at different spatial scales. The potential interaction between the genetic architecture and spatial scale of adaptation means that the identities of adaptive loci may vary across the species range, potentially affecting the rate and manner in which populations will evolve in response to warming (Kardos & Luikart, 2021; Oomen et al., 2020). Investigating these factors in parallel will improve the predictions of evolutionary responses to climate change.

## 3 GENOMIC REACTION NORMS FROM COMMON‐GARDEN‐GRADIENT EXPERIMENTS CAN HELP IDENTIFY THE GENETIC BASIS AND SPATIAL SCALE OF VARIABLE RESPONSES TO ENVIRONMENTAL CHANGE

### 3.1 Common‐garden‐gradient experiments inform plastic and evolutionary responses

Reaction norms are typically constructed using common‐garden or reciprocal transplant experiments whereby the same set of genotypes are exposed to multiple environmental conditions in a controlled setting, such that phenotypic variation among genotypes, while accounting for potential maternal effects, must have a genetic basis. Because a single individual can only experience one environmental condition while maintaining identical developmental histories as others, reaction norms can be quantified for a single genotype in clonal species, or at the family, population or species levels in others (Oomen & Hutchings, 2020). This distinguishes reaction norms somewhat from ‘performance curves’, which can also be based on repeated measures of the same individuals (Schulte et al., 2011). Therefore, reaction norms along a temperature gradient are a specific type of ‘thermal performance curve’, a very common concept in physiology (Schulte, 2015; Schulte et al., 2011). The common‐garden‐gradient reaction norm approach has been used for decades to quantify plastic and evolved responses to temperature in a wide variety of taxa, including plants, invertebrates and vertebrates, especially fishes (Hutchings, 2011; Murren et al., 2014; Oomen & Hutchings, 2015; Sultan, 2017).

### 3.2 Reaction norms can be constructed for genes and genomes

While the use of genomic reaction norms to study the expression of a select number of candidate genes in an individual manner has gained some traction (Aubin‐Horth & Renn, 2009; Croisetière et al., 2010; Smith et al., 2013), their potential to study genome‐wide variation in gene expression is still under‐realized in ecological contexts (but see Lafuente & Beldade, 2019). Genes do not operate in isolation, but rather form a network of additive effects and epistatic interactions that can promote or constrain adaptation (Satokangas et al., 2020). Many traits (particularly those related to climate) are associated with hundreds to thousands of loci (Bay et al., 2017) and/or large blocks of loci in tight linkage disequilibrium (Oomen et al., 2020). Therefore, a lack of a holistic framework for analysing the reaction norms of all genes in a collective manner limits our understanding of the molecular and physiological underpinnings of responses to the environment. There is also a need for the development of bioinformatic tools to facilitate the interpretation of genomic reaction norms and the insights gained from vast amounts of genomic data. By mechanistically linking DNA sequence and transcript abundance to downstream phenotypes and fitness under a range of environmental conditions, genomic reaction norms can reveal the genetic basis of climate responses and evolutionary constraints on climate adaptation (Oomen & Hutchings, 2017; Rivera et al., 2021).

Transcriptomics provides a key bridge in this regard, by linking genes to phenotypes at a genome‐wide scale. Because of its presumed influence on physiological, morphological, behavioural and life‐history traits, gene expression is considered a putatively adaptive phenotype itself. Expression profiles can, therefore, be used to inform the design of conservation units (Hansen, 2010; Vandersteen Tymchuk et al., 2010) and biomarkers for sublethal stress (Akbarzadeh et al., 2018) as well as to monitor the condition of wild individuals (reviewed by Evans & Hofmann, 2012). In the context of the experimental framework outlined above, transcriptomics enables the construction of genomic reaction norms on a genome‐wide scale. Genomic reaction norms can be used to characterize the molecular basis of phenotypic plasticity and identify candidate genetic variants underlying adaptive phenotypes, thus adding important pieces to the genotype–phenotype‐environment puzzle (Aubin‐Horth & Renn, 2009; Shama et al., 2016).

## 4 EXPERIMENTAL AND ANALYTICAL FRAMEWORK FOR GENOMIC REACTION NORMS

### 4.1 Common‐garden experimental design

General wisdom for common‐garden experimental design (Oomen & Hutchings, 2015; Schulte et al., 2011; van de Pol, 2012) and gene expression experiments (Fang & Cui, 2011; Hansen, Wu, et al., 2012; Oomen & Hutchings, 2017; Sendler et al., 2011) has been covered elsewhere. Rivera et al. (2021) reviewed methods to understand the role of gene expression plasticity in stress tolerance using a genomic reaction norm framework. Here, we focus on some issues of particular relevance for genomic climate reaction norms.

**FIGURE 2:**
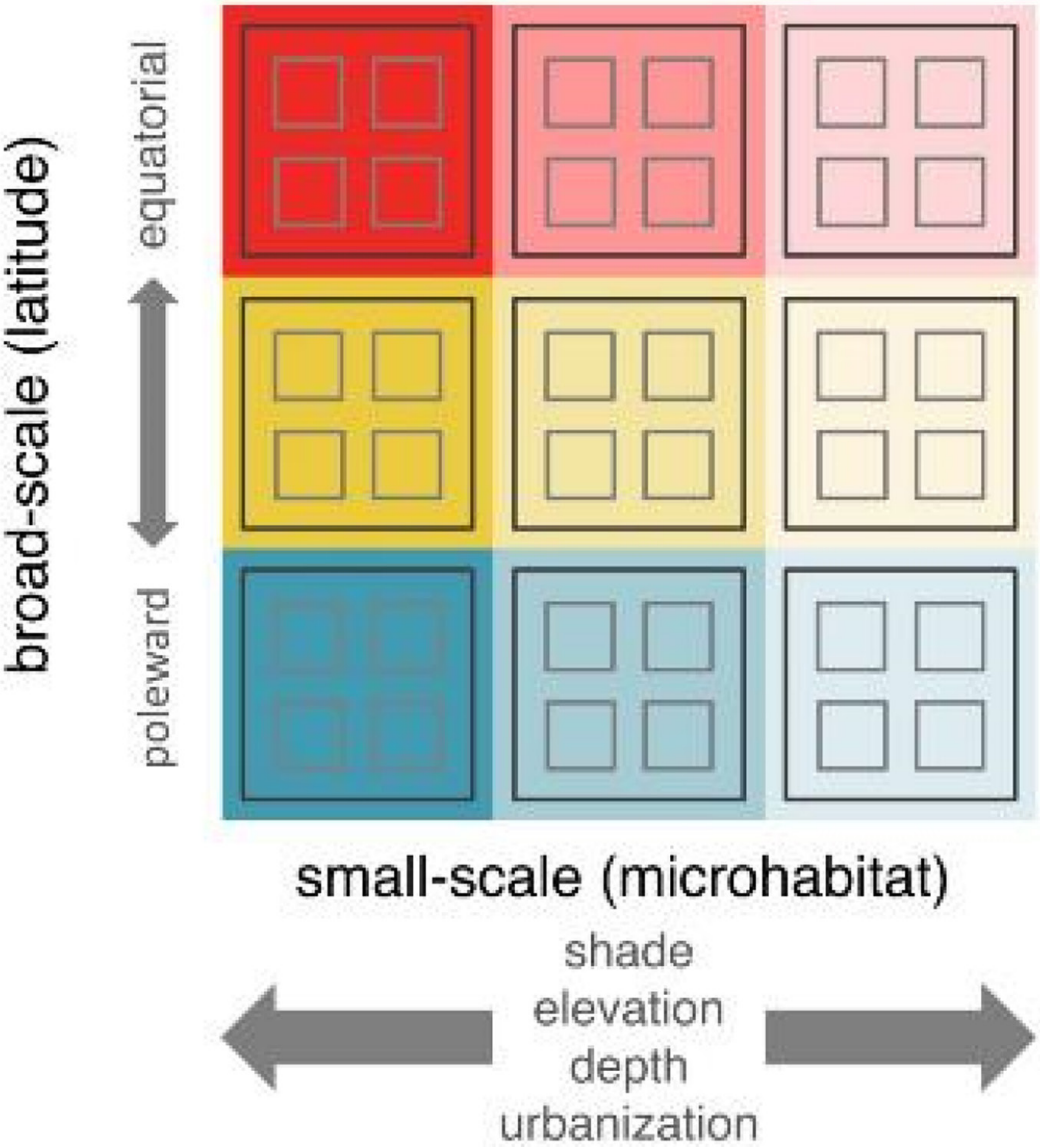
Example 3 × 3 common‐garden experimental design across two scales of biological variation, wherein local adaptation to temperature across broad (latitudinal) scales and small (microhabitat) scales are assessed. Black squares represent broad‐scale × small‐scale groups, and grey squares represent experimental replicates (e.g. plots or tanks). In many cases, it will not be feasible to assess all groups at the same time, in which case systematic temporal overlap is recommended to estimate potential batch effects (*B*)

It is usually desirable to expose genotypes to a range of environments encompassing native and projected values, according to the IPCC (IPCC, 2018) or more local projections based on in‐house simulations. However, at least three environmental treatments are required in order to distinguish linear and nonlinear reaction norms (Figure 1). For reasons discussed in the previous section, depending on a species distribution range, dispersal capabilities, reproductive barriers, scale of disturbance and putative genomic architecture of adaptation (if known), it might be relevant to investigate variation across one or more levels of biological variation (e.g. strain, family, population, species) and/or spatial scales (e.g. microhabitat, local, regional) to understand the genomic basis and spatial scale of adaptation (Figure 2).

It is critical to link genomic reaction norm variation to fitness (herein considered as lifetime reproductive success) in order to understand its impact on demographic rates, which are of ultimate interest in biodiversity conservation and management. Progress can be made by correlating reaction norm variation between genomic and downstream fitness‐related phenotypes, especially reproductive success, survival and, in the context of climate change, metabolism, growth and thermal tolerance, among others. Even for conventional common‐garden experiments, it is challenging to link any particular trait to fitness because: (a) there are a near‐infinite number of traits (both known and unknown) and times at which they can be measured, (b) many traits are tightly correlated and (c) these relationships are often environmentally dependent. Therefore, we are limited to likelihoods and intuition to conclude which traits are relevant for the cumulative survival and reproductive success over an individual's lifetime. These challenges are in some ways exacerbated for genomic reaction norms because the expression of many genes is extremely dynamic on even very short time‐scales, reflecting an instantaneous response to environmental conditions. This means that the chance of capturing a moment of gene expression that is highly relevant for fitness is less likely. On the other hand, constitutive differences in gene expression among genotypes may be more confidently linked to fitness, given their consistency throughout life. To mitigate these challenges, expression can be measured during (or immediately preceding) key mortality and reproductive events, such that environment and genotype × environment effects can be most strongly associated with these core fitness traits (e.g. Harder et al., 2020). Measuring expression across environmental gradients (and ideally at multiple time points) can identify constitutive differences in expression attributable to genotype that are associated with downstream differences in fitness among genotypes. Rivera et al. (2021) provide a framework for linking gene expression plasticity and fitness from the perspective of comparing stress‐tolerant and susceptible populations.

It is a standard practice to sample several individuals (i.e. biological replicates) from multiple treatment replicates (e.g. garden plots, jars, tanks) in order to compare phenotypic variation within and between treatments (e.g. using pairwise comparisons or ANOVA statistics). With regard to sample size, genomic reaction norms differ from those of traditional phenotypes because fewer individuals are needed to achieve sufficient power for the most common types of differential expression analysis. Although it depends on the amount of biological variation in the study system (which can be estimated using a pilot study and statistical power calculator), the analyses of gene expression estimate within‐gene variance using information on the expression levels of all genes and achieve sufficient power with fewer biological replicates (Todd et al., 2016). Therefore, it will often be practical to sample more individuals for traditional phenotypic measurements and only perform gene expression analyses on a subset of these.

### 4.2 An—analytical framework for high‐dimensional genomic reaction norms

After gene expression data generation and exploration (see Box 1 for details), differential expression analysis can be used for genomic reaction norm construction. The inherent complexity of differential expression analysis, which quantifies the mean relative expression levels of each of thousands of genes between the groups, originally necessitated simple experimental designs such as pairwise contrasts. Now it is possible to construct multifactorial mixed‐effect models to analyse the influences of genotype, environment and genotype × environment interactions on gene expression while accounting for random variation due to experimental replicate or sequencing run/lane (i.e. random effects, collectively known as ‘batch’ effects [*B*] in differential expression analysis).

BOX 1 **Gene expression data generation and exploration for genomic reaction norm construction**

Genomic reaction norms can be constructed using traditional species‐specific microarrays (Meier et al., 2014; Morris et al., 2014), high‐throughput RNA sequencing (Casasa et al., 2020; Wellband & Heath, 2017) or both (Huang et al., 2020). The choice will largely depend on the genomic resources available for a particular species (i.e. if a relevant microarray is available) and budget considerations, as RNA sequencing is more costly per individual library (but note that samples can be pooled; for a detailed comparison, see Oomen & Hutchings, 2017). Ultimately, the base‐pair resolution of RNA sequencing will make it the optimal choice in many instances (Oomen & Hutchings, 2017; Ozsolak & Milos, 2011; Wang et al., 2009). One particular advantage of RNA sequencing is that it enables variant calling on the same individuals (Lopez‐Maestre et al., 2016; Piskol et al., 2013), such that the effects of different variants and genomic arcitectures on gene expression can be readily evaluated (see Section 4.3).

RNA expression, integrity and sequencing are extremely sensitive to time and intrinsic and extrinsic environmental factors. The greatest systematic source of error in RNA sequencing experiments is typically that imposed by different sequencing lanes or runs (Leek et al., 2010; Leigh et al., 2018). Initial exploratory analyses are extremely useful for identifying such unintended sources of variation prior to hypothesis testing. Typical RNA‐seq workflows involve initial data exploration, using tools that summarize variation along two visible dimensions, such as multidimensional scaling (MDS), biological coefficient of variation (BCV), principal component analysis (PCA) and t‐distributed stochastic neighbour embedding (tSNE) plots, implemented in various R packages [e.g. edgeR (Chen et al., 2014), DEseq (Love et al., 2014) and Rtsne (van der Maaten, 2014; van der Maaten & Hinton, 2008)]. However, for experiments of the complexity discussed herein, involving several axes of known and unknown variation, a gene‐by‐gene partitioning of variance can be helpful, as implemented in the R package variancePartition (Hoffman & Schadt, 2016). Grade of Membership (GoM) models [known to population geneticists as STRUCTURE plots (Pritchard et al., 2000)] are also useful for assessing the dominant drivers of gene expression variation based on expression clusters rather than individual genes, implemented in CountClust (Dey et al., 2017).

This non‐exhaustive list of approaches should facilitate the construction of a model that appropriately captures important sources of technical and biological variation in gene expression. Such models can be fit to transcript abundance data and used to test for differential expression using, for example, the r package edger (Chen et al., 2014) together with limma (Law et al., 2016; Ritchie et al., 2015).

A basic model resembles the formula for variance components of a quantitative trait, whereby *V*_*Px*_ is the variance in expression of each gene, *V*_*G*_ is the variance in expression due to genotype, *V*_*E*_ is the variance in expression due to environment, *V*_*G×E*_ is the variance in expression due to the interaction between genotype and environment, *B* represents batch effects and *Ɛ* represents error:

(1) $$V_{\mathrm{Px}}=V_{G}+V_{E}+V_{G\times E}+B+Ɛ.$$

A list of differentially expressed genes is obtained for each model term separately, depending on whether that factor significantly affected transcript abundance overall. Unlike a typical reaction norm model based on a single phenotype, thousands of gene expression phenotypes are assessed simultaneously, and therefore different model terms might explain a significant amount of expression variation for different genes. If a model term does not affect differential expression of any genes, it is justified to remove it from the model. However, whether to retain a model term affecting expression of only one or few genes is a judgement informed by the specific research question and planned downstream analyses.

**FIGURE 3:**
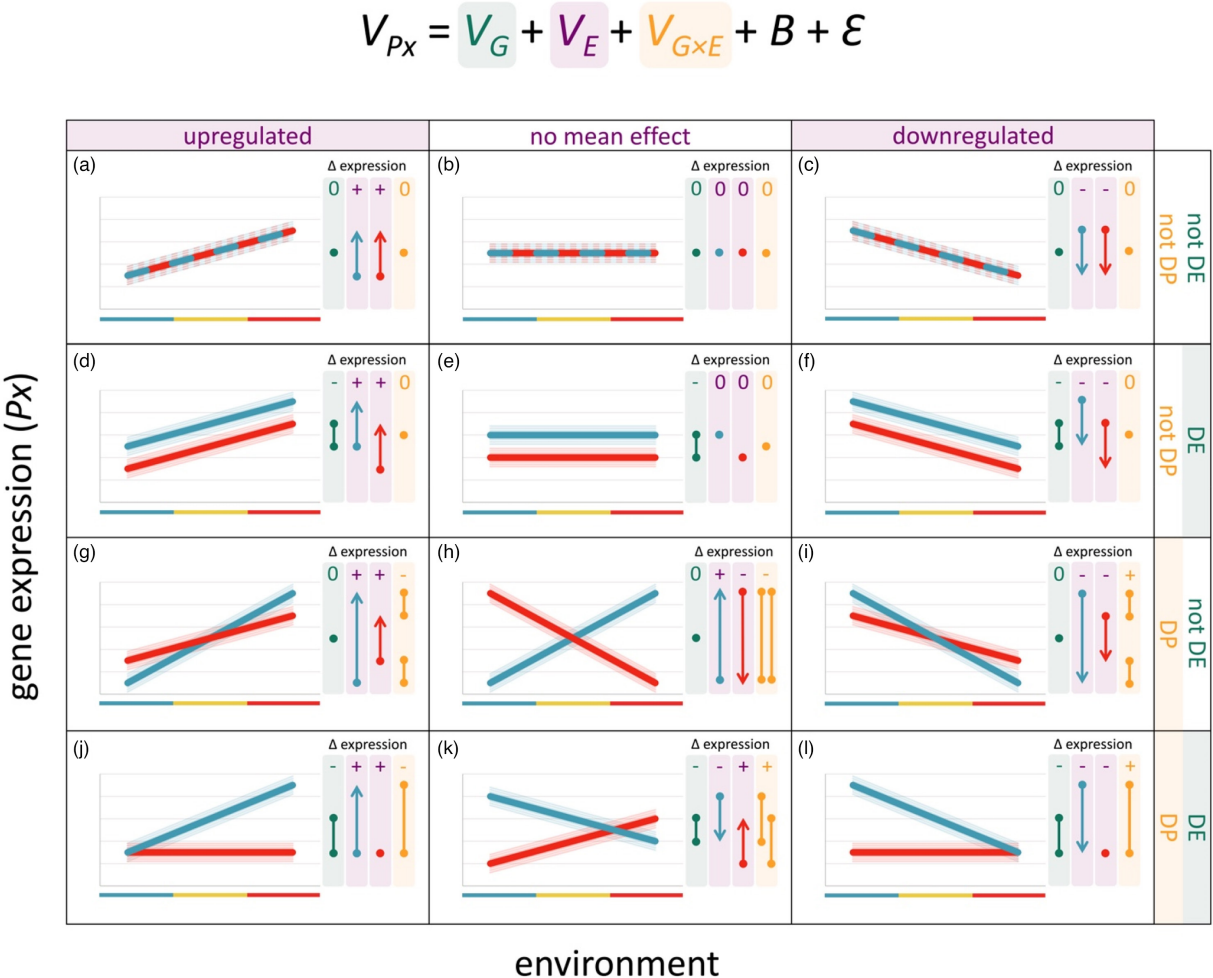
A reaction norm framework for decomposing gene expression (*V*_*Px*_) differences into overall effects of genotype (*V*_*G*_; green), environment (*V*_*E*_; purple) and genotype × environment interaction (*V*_*G×E*_; orange; Equation 1 fixed effects). For each individual gene, there are 12 categories (a‐l) of gene expression patterns along an environmental gradient. The difference (Δ) in gene expression attributable to each component (i.e. model term) in each category is summarized in terms of direction and relative magnitude compared to the selected model intercept. In this example, the intercept is set to the blue genotype and the blue environment (e.g. the poleward genotype and the lowest temperature; Figure 2). Non‐zero differences in overall expression are categorized as positive (+) or negative (−) in direction relative to the model intercept. Relative magnitudes of expression differences are reflected in the length of arrows and segments below these signs. Arrows represent purely environmental effects (*V*_*E*_), which are described as up‐ or downregulated along an environmental gradient, separately for each genotype. Segments represent effects with a genetic component (*V*_*G*_ and *V*_*G×E*_). *V*_*G*_ is referred to as differentially expressed (DE) between genotypes with respect to mean expression across all environments. *V*_*G×E*_ is referred to as differentially plastic (DP) between genotypes with respect to their responses to the environment. If significant overall effects are found, one can conduct post hoc contrasts between specific levels of the factor to determine where the differences lie

In the context of genomic reaction norms, we propose the terms ‘differentially expressed’, ‘plastic’ and ‘differentially plastic’ to describe genes whose expression is significantly affected by *V*_*G*_, *V*_*E*_ and *V*_*G×E*_ respectively (Box 2; Figure 3). Categories aid in genomic reaction norm interpretation and provide groups of genes with important distinctions for use in downstream analyses. Importantly, they are independent of whether gene expression is, on average, upregulated, downregulated or unchanged in relation to the environmental gradient (Figure 3). Thus, one can extract subsets of genes according to both of these criteria and discuss them accordingly.

BOX 2 **A high‐dimensional genomic reaction norm framework**

By linking differential expression model outputs with corresponding reaction norms, this genomic reaction norm framework facilitates: (a) disentangling genetic and environmental components of gene expression variation, (b) interpreting expression difference magnitudes and directions and (c) categorizing genes according to similar expression patterns (Figure 3). In pairwise contrasts common to traditional differential expression analyses, one level of a factor is selected as the reference against which the second level is compared. In mixed‐effects models described here (Equations 1–3), the level of each factor that comparisons are made against (the ‘model intercept’) must be set in order to identify the direction of mean differential expression between the groups (for more on model intercepts and mixed‐effects models in general, refer to Harrison et al., 2018).

For example, in Figure 3k, differential expression is negative because the red genotype has lower mean expression compared to the blue genotype that is set as the intercept. However, differential plasticity is positive because the red genotype has a more positive slope compared to the blue genotype. In contrast, in Figure 3h, differential plasticity is negative because the red genotype has a more negative slope compared to the blue genotype. For the examples illustrated in both categories (Figure 3h,k), differential plasticity is equal to two times the magnitude of each slope because the slopes are equal with opposite signs (indicated with two orange segments of equal length to the *V*_*E*_ effect arrows). For the other categories illustrating differential plasticity (Figure 3g,i,j,l), its magnitude is simply the difference between the magnitudes of each slope because the slopes have the same sign.

This framework refers to the overall effects of *V*_*G*_, *V*_*E*_ and *V*_*G×E*_ on gene expression. After obtaining a significant effect for a factor, one can conduct post hoc contrasts between specific levels to identify where significant differences lie. For such contrasts, the intercept is set for the desired genotype and environment one wishes to compare against.

For simplicity, we present a comparison of only two of the ‘groups’ represented in Figure 2 [poleward (blue) vs. equatorial (red) at low ‘microhabitat’ values]. It is reasonable to assume that intermediate spatial scales (e.g. yellow environment) would produce intermediate patterns of adaptation. However, ideally one would incorporate intermediate scales as they could represent transition zones with unique properties (e.g. contact or hybrid zones). This framework could also be expanded to incorporate a nested spatial scale (e.g. microhabitat; Figure 2) using a nested model of phenotypic variance (Equation 2) and *z*‐axis to produce three‐dimensional reaction norms. This framework is also applicable to other types of ‘omic’ data (e.g. proteomics, phenomics).

For example, one might be interested in genes that show a mean increase in expression in response to warmer temperatures (Figure 3a,d,g,j), or for which the degree of increase depends on genotypes adapted to different temperatures (Figure 3g,j), whether (Figure 3j) or not (Figure 3g) the genotypes differ in mean expression. These classifications could apply to reaction norms in general. However, rarely is information on so many phenotypes available that such categories would become useful, if not essential, for biological interpretation (although this is likely to change in the burgeoning era of ‘phenomics’ borne from applying machine learning to big datasets to extract phenotypic information *en masse*; Lürig et al., 2021). Downstream analyses and interpretation are also often performed separately for up and downregulated genes. The framework herein provides a means of doing so according to genotype, environment, their interaction or any combination thereof.

The utility of such categories of differentially expressed genes is evident in a study comparing genomic reaction norms among northern and southern populations of damselfly *Ischnura elegans* across their summer temperatures to determine whether climate adaptation is attributable to the plasticity or evolution of gene expression (Swaegers et al., 2020). Because most differentially expressed genes showed fixed differences in mean expression between the populations (i.e. *V*_*G*_; Figure 3d–f), it was concluded that changes in constitutive gene expression have driven, and will presumably continue to drive, thermal adaptation in this species. Of those genes exhibiting a plastic response to temperature but a lack of differential plasticity (Figure 3a,c,d,f), more than half were upregulated (Figure 3a,d). Interestingly, few of these genes overlapped with those associated with the response to short‐term heat stress (Lancaster et al., 2016), suggesting that short‐ and long‐term responses to warming involve largely different pathways. Many genes also exhibited *V*_*G×E*_ effects (Figure 3e–l). Swaegers et al. (2020) further divided these genes into those that showed a pattern of: (a) genetic compensation (Figure 3j for genes that are, on average, upregulated in response to temperature; flipping the plot vertically would produce the equivalent pattern for downregulated genes), (b) genetic assimilation (Figure 3l for genes that are, on average, downregulated in response to temperature; flipping the plot vertically would produce the equivalent pattern for upregulated genes) or (c) reversal (Figure 3h,k). The most abundant category was genetic compensation, suggesting that this process is the most likely to drive the evolution of plasticity in response to mild warming in the northern population. In this way, the use of such categories of genomic reaction norm variation (or specifications and combinations thereof) enabled the tests of a variety of hypotheses regarding evolutionary adaptation to climate change.

### 4.3 Adding spatial and genomic‐architectural complexity to the genomic reaction norm framework

Further insight into the spatial scale and genomic basis of adaptation can be gained from more complex experimental designs. In the context of examining genomic reaction norms at multiple levels of biological organization or spatial scales (Figure 2), one can extend the model (1) to nest multiple levels/scales, wherein *V*_*Gi*_ is the smaller/lower scale/level and *V*_*Gj*_ is the larger/upper one:

(2) $$V_{\mathrm{Px}}=V_{\mathrm{Gi}}\left(V_{\mathrm{Gj}}\right)+V_{E}+V_{\mathrm{Gi}\times E}\left(V_{\mathrm{Gj}\times E}\right)+B+Ɛ.$$

Alternatively, one can perform single nucleotide polymorphism (SNP) and structural variant (SV) calling on the transcriptome sequence data to characterize population structure according to different genomic architectures, which often differs between SNPs and SVs (Lopez‐Maestre et al., 2016; Mérot et al., 2020; Norman et al., 2014; Piskol et al., 2013). Doing so permits comparison of the effects of different genomic architectures on genomic reaction norm variation, such as subpopulations determined from genome‐wide SNP variation (*V*_*Gsnp×E*_) and known SVs (*V*_*Gsv×E*_) and their respective interactions with the environment (*V*_*Gsnp×E*_ and *V*_*Gsv×E*_):

(3) $$V_{\mathrm{Px}}=V_{\text{Gsnp}}+V_{\mathrm{Gsv}}+V_{E}+V_{\text{Gsnp}\times E}+V_{\mathrm{Gsv}\times E}+B+Ɛ.$$

Disentangling the effects of sequence and structural variation in the genome is important for understanding the spatial distribution of adaptive variants and the evolutionary potential of populations to adapt to environmental change (Mérot et al., 2020; Oomen et al., 2020).

### 4.4 Functional interpretation of genomic reaction norm variation

Functional analyses of gene expression variation depend on many factors, such as the numbers of genes exhibiting significant variation in expression and the genomic resources available (e.g. high‐quality reference genome and annotation), and are discussed in depth elsewhere (e.g. Alvarez et al., 2015; Oomen & Hutchings, 2017; Ozsolak & Milos, 2011; Todd et al., 2016). The analyses of particular relevance in the context of genomic reaction norms are: (a) functional enrichment tests to infer which molecular and physiological processes vary [e.g. using AmiGO (Carbon et al., 2009) or PANTHER (Mi et al., 2019)], (b) expression Quantitative Trait Loci (eQTLs) to pinpoint the genomic basis of gene expression variation (Huang et al., 2020; Lafuente & Beldade, 2019; Majewski & Pastinen, 2011), (c) allele‐specific expression (Khansefid et al., 2018) and patterns of alternative splicing (Engström et al., 2013; Verta & Jacobs, 2022) from RNA‐seq data to reveal more detailed, gene‐specific phenomena relevant for particular species' responses to the environment and (d) weighted gene co‐expression network analysis (WGCNA; Langfelder & Horvath, 2008) to infer plastic and evolved differences in gene regulatory networks (Casasa et al., 2020, 2021; Huang et al., 2020; Rose et al., 2016) and functions of unknown genes (Orsini et al., 2018; Stanford & Rogers, 2018). Note that weighted gene co‐expression network analysis decomposes genome‐wide expression patterns into co‐expression modules that can be similarly integrated into a linear modelling framework for partitioning of phenotypic variance (Aubin‐Horth & Renn, 2009). This reduction of dimensionality prior to genomic reaction norm construction provides an adjusted approach to the high‐dimensional one described herein and is sufficient for describing dominant patterns of gene expression.

Finally, links between genomic reaction norm variation and fitness can be explored genome‐wide by correlating the expression patterns of genes or gene co‐expression networks with fitness‐related phenotypes. A similar approach was used to correlate genes differentially expressed between fish developmentally and transgenerationally exposed to warmer temperatures with metabolic rate and aerobic scope to understand the molecular basis of plastic responses to climate change (Veilleux et al., 2015). Harder et al. (2020) coupled genomic reaction norms (thiamine treatment vs. control) with formal survival analyses of early life stages of Atlantic salmon *Salmo salar*. In doing so, they identified the genetic basis of family‐level variation in survival in response to thiamine deficiency, which is required for an adaptive response to this emerging stressor. See the section *Common‐garden experimental design* for more discussion on linking gene expression to phenotypes and fitness.

## 5 GENOMIC REACTION NORMS CONTRIBUTE TO A HOLISTIC FRAMEWORK FOR FORECASTING CLIMATE CHANGE RESPONSES

Thus far, we have discussed how genomic common‐garden‐gradient experiments are powerful approaches for studying both plastic and adaptive responses to environmental change and that the reaction norm analytical framework facilitates interpretation of such experiments. In this section, we will discuss the potential of a holistic approach, which combines genomic, phenotypic and fitness data with eco‐evolutionary modelling of population and ecosystem dynamics, for predicting changes in global biodiversity in response to anthropogenic change.

The interplay between ecology, evolution and genomics represents a nexus of research endeavours with tremendous potential to lead to paradigm shifts in long‐standing hypotheses and theories of how organisms interact with and are adapted to their physical and biological environments. Notwithstanding the tremendous strides that have been realized in understanding the functional genomic basis underlying traits of potential or known ecological or evolutionary importance (e.g. Barson et al., 2015; Jones et al., 2012; Wellenreuther & Bernatchez, 2018), there remain major research gaps in linking ecology, evolution and genomics across different scales of biological organization (Coulson et al., 2017; Kuparinen & Hutchings, 2017; Oomen & Hutchings, 2017). The environmental influence at every level—whether by stimuli integrated at the cellular level to influence downstream phenotypes, selection pressures acting on phenotypes to alter the genetic makeup of subsequent generations or indirectly through ecosystem interactions—necessitates considering the dynamics of the system as a whole in order to understand variability in its components.

While the need for a more holistic perspective on environmental responses has been realized for some time, a lack of sufficient data and analytical tools has, to some extent, hindered progress in this regard (reviewed by Pacifici et al., 2015, albeit without consideration for the role of genomics). Coulson et al. (2017) and Bay et al. (2017) made particular conceptual progress for the integration of genomics into predictive modelling of evolutionary responses to environmental change. Bay et al. (2017) laid out four components of the ‘evolutionary response architecture’: (a) population dynamics, (b) genetic architecture, (c) the spatial distribution and abundance of adaptive alleles and (d) phenotypic plasticity. As implied by this list, understanding behavioural responses, such as dispersal, and other potential manifestations of plasticity, is key to predicting evolutionary responses. While an evolutionary perspective on environmental change is undoubtedly vital in the long term, a strategy that integrates predictions of these short‐term, non‐evolutionary responses will improve the accuracy of longer term predictions while providing highly practical knowledge about tangible outcomes for policymakers. A holistic response architecture, composed of behavioural, plastic and evolutionary responses and their underlying, interconnected components, provides the best opportunity for successful wildlife management under climate change.

Genomic reaction norms have a key role to play in informing such models. When linked to fitness, they can inform population‐specific plastic responses to short‐term changes in climate by revealing the extent of local adaptation in plasticity. Reaction norms for plasticity enable integrating ecological processes across scales from individuals to populations, as demonstrated in a hierarchical Bayesian statistical framework for forecasting regional‐scale population dynamics (Lasky et al., 2020). Furthermore, genomic reaction norms inform evolutionary responses of mean phenotypes and their plasticities by identifying their underlying genomic architectures.

Genomic trait architecture is vital to evolutionary forecasts of climate change responses, including adaptive trait evolution and extinction risks (Bay et al., 2018; Kardos & Luikart, 2021; Layton et al., 2021; Oomen et al., 2020; Ruegg et al., 2018). Reaction norms and genomic architecture can also aid in predicting range shifts, when combined with knowledge of the dispersal potential and connectivity of populations (Lasky et al., 2020).

Identifying the genomic basis of plasticity itself (e.g. transcription factors, ‘hub’ genes; Costanzo et al., 2010) will also improve quantitative predictions of its diversity and effects. Although metrics of genetic diversity (e.g. allelic richness, heterozygosity) have been used considerably for identifying vulnerable populations (Lande & Shannon, 1996; Storfer, 1996), the quantification of genetic diversity in plasticity lacks similar metrics. Quantifying plasticity and its diversity in natural populations is logistically challenging given the poor feasibility of large‐scale common‐garden experiments for many organisms. Identifying genetic markers for plasticity of key traits would enable its characterization on broad geographic scales in diverse natural systems, though not without challenges surrounding validation. Nonetheless, establishing explicit metrics for comparing genetic variation in reaction norm slopes and shapes among populations and species would represent a step towards detecting general patterns of plasticity and ‘laws’ surrounding its occurrence.

The quantitative genetic approach of decomposing phenotypic variance (including for gene expression) using reaction norms facilitates connections between the population and ecosystem levels through integration with food web modelling (Barbour & Gibert, 2021). Changes in the relative contributions of *V*_*G*_, *V*_*E*_ and *V*_*G×E*_ under climate change can alter the number and magnitude of species interactions, thereby rewiring food web structure and stability (Barbour et al., 2016; Gibert & DeLong, 2017). Each of these potential rewiring effects might be predictable (Barbour & Gibert, 2021). Climate change might decrease *V*_*G*_ through selection, leading to mismatches in the phenotypic variability of interacting species and therefore fewer, yet stronger, species interactions. Conversely, climate change might increase *V*_*G*_ by reducing barriers to gene flow between the populations, which is expected to produce more, weaker interactions. Increases in *V*_*E*_ or *V*_*G×E*_ are also predicted to produce more, weaker interactions, but at a faster pace. Decomposition of *V*_*P*_ using genomic reaction norms holds great promise for testing these predictions and revealing the nature and pace of community‐ and ecosystem‐level effects from climate change impacts on population‐level phenotypes.

More broadly, the integration of genomic data into predictive modelling of responses to climate change is a ripe field for future research. Beyond its academic utility, modelling demonstrates how abstract processes, such as local adaptation and human‐mediated evolution, affect the food we eat, the resource‐based economies on which we thrive and the suite of ecosystem services that nature provides. Thus, such models would be of direct value for wildlife managers and conservation authorities, but would also translate eco‐evolutionary dynamics of complex biological systems in a changing world into tangible outcomes for the public and policymakers. Genomic reaction norms can contribute several pieces to this puzzle of prediction.

## CONFLICT OF INTEREST

The authors have no conflict of interest to declare.

## AUTHORS' CONTRIBUTIONS

R.A.O. conceived the ideas; R.A.O. wrote the manuscript with critical input from J.A.H.; Both authors approved the original submission for publication. R.A.O. approved the revised submission for publication on behalf of both authors.

## Data Availability

No original data were presented in this article.
